# Ensemble averaging deep neural network for botnet detection in heterogeneous Internet of Things devices

**DOI:** 10.1038/s41598-024-54438-6

**Published:** 2024-02-16

**Authors:** Aulia Arif Wardana, Grzegorz Kołaczek, Arkadiusz Warzyński, Parman Sukarno

**Affiliations:** 1 Wrocław University of Science and Technology, Wrocław, Poland; 2 Telkom University, Bandung, Indonesia

**Keywords:** Anomaly detection, Ensemble averaging, Internet of things, Intrusion detection, Neural network, Computer science, Information technology

## Abstract

The botnet attack is one of the coordinated attack types that can infect Internet of Things (IoT) devices and cause them to malfunction. Botnets can steal sensitive information from IoT devices and control them to launch another attack, such as a Distributed Denial-of-Service (DDoS) attack or email spam. This attack is commonly detected using a network-based Intrusion Detection System (NIDS) that monitors the network device’s activity. However, IoT network is dynamic and IoT devices have many types with different configurations and vendors in IoT environments. Therefore, this research proposes an Intrusion Detection System (IDS) by ensemble-ing traffic from heterogeneous IoT devices. This research proposes Deep Neural Network (DNN) to create a training model from each heterogeneous IoT device. After that, each training model from each heterogeneous IoT device is used to predict the traffic. The prediction results from each training model are averaged using the ensemble averaging method to determine the final result. This research used the N-BaIoT dataset to validate the proposed IDS model. Based on experimental results, ensemble averaging DNN can detect botnet attacks in heterogeneous IoT devices with an average accuracy of 97.21, precision of 91.41, recall of 87.31, and F1-score 88.48.

## Introduction

Recently, the development of the IoT environment has become extensive and complex in structure. Along with this advancement, the occurrence of cyber-attacks has also increased in complexity^1^. Among these attacks, botnet attacks have proven to be a valuable tool for taking control of IoT devices to launch another attack or steal sensitive information from the device. Botnets use networks to spread quickly and increase the possibility of infecting many IoT devices^2^. NIDS commonly monitors the whole network by combining traffic from each host to analyze anomalies on the network^3^. However, the IoT environment has heterogeneous devices with different configurations, vendors, and types^4^. Detecting anomalies in the different characteristics of the host in the network is challenging as the traffic pattern differs in each heterogeneous IoT device^5^. To overcome that problem, an ensemble-based NIDS to detect cyberattacks in an IoT environment is proposed. According to research^6^, ensemble learning is creating subset training to produce a subset classifier for better result prediction by combining diversity among the training models. Analyzing data among diverse behavior in ensemble learning can help NIDS identify broader attack patterns in IoT networks with heterogeneous devices.

The NIDS commonly uses machine learning to analyze network traffic for anomaly detection. One of the machine learning algorithms that is famous for anomaly detection is DNN. The algorithm helps learn complex patterns and features from data^7^. On the other hand, ensemble averaging is a highly advantageous ensemble learning method due to its ability to merge the strengths of many diversity classifiers. The ensemble averaging technique can produce more precise and resilient results by averaging their predictions. This approach can mitigate the imperfections of individual classifiers and attain better detection performance, such as increased detection rates and decreased false positive rates^8,9^. The NIDS can best detect botnet attack patterns in heterogeneous IoT devices by leveraging a combination between DNN and ensemble averaging for anomaly detection.

This research used the N-BaIoT dataset from research^10^ to simulate botnet attacks and heterogeneous IoT devices. This research conducts a more in-depth study on comparing the performance of prediction results between a single training model and averaging training model in each heterogeneous IoT device.

### Research questions

This research begins by defining research questions, which include:

1. How is the performance of NIDS for botnet detection using a DNN in each IoT device?
2. How is the performance of NIDS for botnet detection using DNN when analyzing traffic from each other IoT devices?
3. How is the performance of NIDS for botnet detection using ensemble averaging DNN when analyzing traffic from each other IoT devices?

### Overview of the paper

This research is organized as follows:

In “Introduction” section explains the background and state-of-the-art of this research. In “Related terms and works” section explains related terms about NIDS in IoT, DNN, and ensemble averaging. The section also explains the contribution and difference between this research with other research. In “Methodology” section explains step-by-step methods to propose the model and evaluation scenario. In “Result and discussion” section discusses the experiment result from the proposed model. In “Conclusion” section concludes this research.

## Related terms and works

This part of the research reviews related terms in DNN, ensemble averaging, and NIDS in IoT. This section also explains related works and contributions from this research.

### DNN

**Figure 1:**
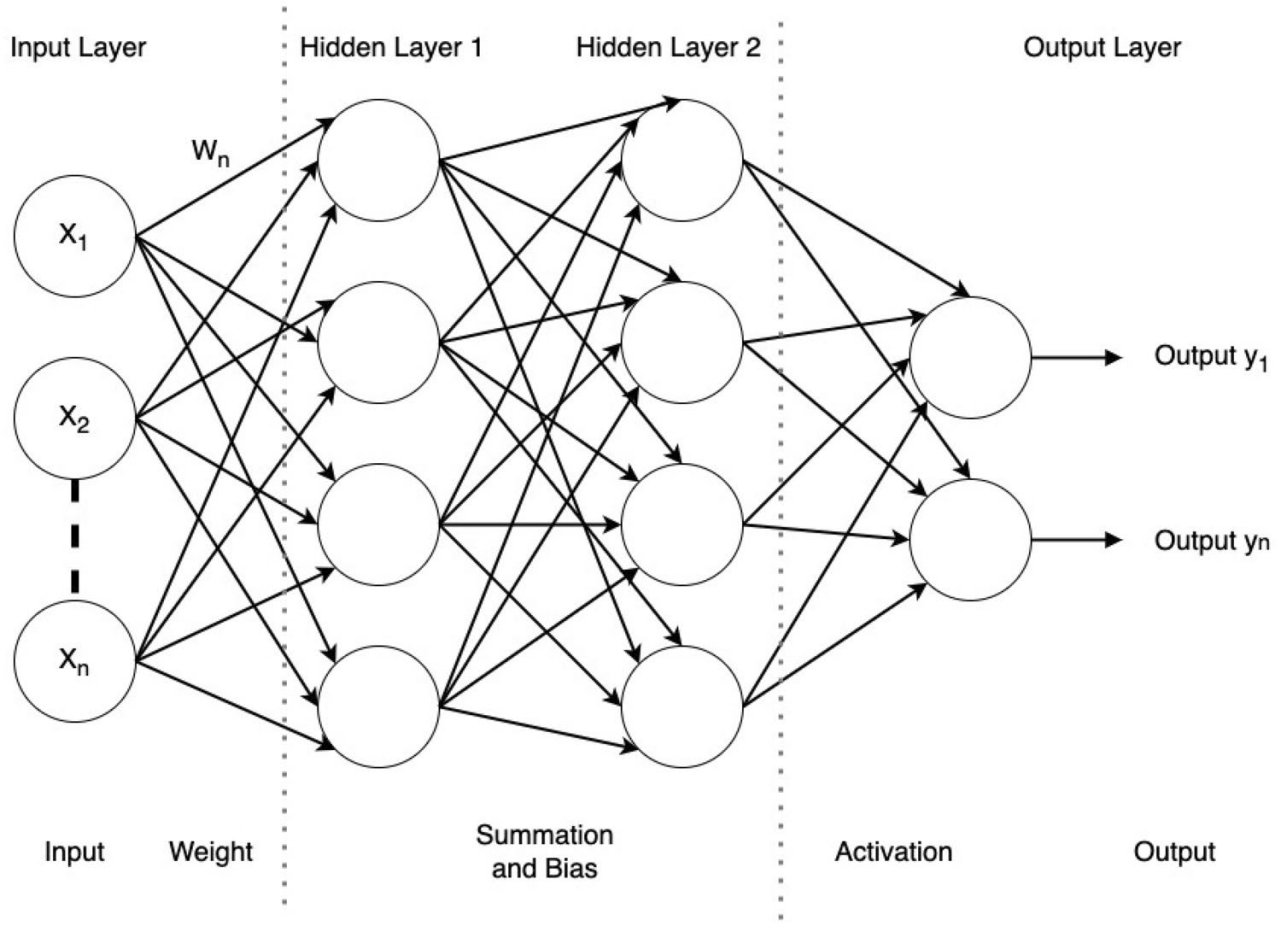
DNN architecture

DNN is a machine-learning algorithm inspired by the human brain. DNN can be represented as three parts consisting of an input layer, an output layer, and a hidden layer^7^. The detail of DNN architecture can be seen in Fig. 1.

The hidden layer processes data input from the input layer into the output system or the output layer. DNN uses more than two hidden layers to process data input. Perceptron is the unit process that processes data in the input, hidden, and output layers. This unit process contains an input value as $x_i$ and a weight value as $w_i$. All input and weight will be processed using summation and adding bias *b* process like Formula 1 in a hidden layer.

1 $$\begin{aligned}{} & {} \sum _{i=1}^{m} (w_{i}, x_{i}) + b \end{aligned}$$

2 $$\begin{aligned}{} & {} R(s) = max (0, s) \end{aligned}$$

3 $$\begin{aligned}{} & {} f(s)_i = \frac{e^{s_i}}{\sum _{j}^{C} e^{s_j}} \end{aligned}$$

The result from the summation and adding bias process continues using activation before producing the output. This research using Rectified Linear Unit (ReLU) activations in hidden layers and the softmax activation function in the output layer. ReLU activations help the model learn complex features and relationships in the data, while softmax in the output layer provides a probabilistic interpretation of the model’s predictions, making it suitable for classification problems. This combination leverages the strengths of both activation functions to create a robust and efficient neural network architecture. The ReLU activation process in the hidden layer can see on Formula 2 and softmax activation process in the output layer can see on Formula 3.

4 $$\begin{aligned} CE = - \sum _{i}^{C}t_ilog(f(s)_i) \end{aligned}$$

This study employs a multi-class classification task to effectively categorize coordinated attacks. In this setup, each sample is assigned to one of the *C* classes. The DNN is configured with *C* output neurons, forming a vector *s* representing scores. The target vector *t* is designed as a one-hot vector, signifying a positive class and $C - 1$ negative classes. Treating the task as a unified classification problem for samples within the *C* classes, the chosen loss function is the Categorical Cross-Entropy loss, given the nature of the multi-class classification. The formula 4 illustrates the Categorical Cross-Entropy loss process during training.

Additionally, this research adopts the Adam (Adaptive Moment Estimation) optimizer for the training process within the DNN. Adam stands out as an optimization algorithm suitable for gradient descent, especially in large-scale problems involving extensive data or parameters. Its efficiency is notable, requiring less memory. Conceptually, Adam combines aspects of both the gradient descent with momentum algorithm and the Root Mean Square Propagation (RMSP) algorithm, as outlined in references^11,12^.

### Ensemble averaging

**Figure 2:**
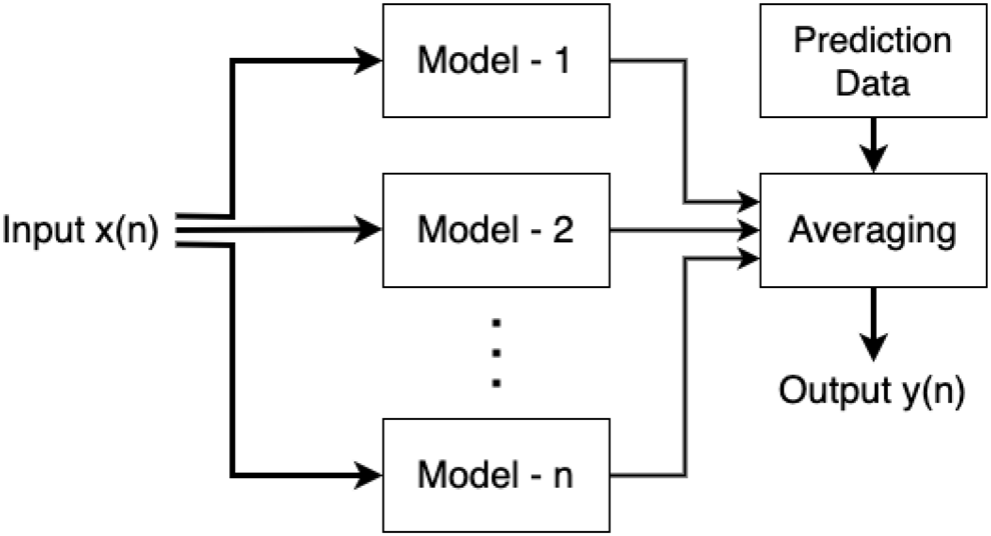
Ensemble Averaging architecture.

The ensemble averaging approach can help tackle the diversity of classifiers by combining the predictions of multiple individual models. The detail of ensemble averaging architecture can be seen in Fig. 2.

Each model is trained on data from a specific source, capturing the unique characteristics of data^8^. By aggregating the predictions of these models through averaging, the ensemble model can capture the diversity of data patterns across different sources. This approach allows the ensemble model to make decisions based on different perspectives, effectively handling the variations in data distributions and patterns across different sources.

5 $$\begin{aligned} y_{AVG} = \frac{1}{n} \sum _{i=1}^{n} y_{i} \end{aligned}$$

Ensemble averaging is simply the average of the prediction result $y_i$ of multiple training models *i*, with $i \in \{1,2, ... , n\}$. The averaging equation of prediction result can be seen in Formula 5^13,14^.

### NIDS in IoT

NIDS is an important part of securing IoT environments. NIDS helps to prevent various threats by monitoring network activity in IoT environments. The devices in IoT networks have limited security features, making them easy targets for attackers. NIDS can help safeguard these devices by proactively detecting and preventing threats before they can damage the device^15^.

NIDS has two main components: a sensor unit to capture and monitor all traffic or activity in a network, and a detector unit to detect anomalies in the network. In IoT environments, each host or IoT device in the network has its own unique network properties, applications, and user behaviors, resulting in variations in activity and traffic patterns. This makes it challenging for the sensor unit to capture heterogeneous traffic from each device. The detector unit also needs the ability to analyze heterogeneous traffic from each device^16^.

### Related works

This research reviews the related literature on the topic of “NIDS for botnet detection in IoT using ensemble learning”. Research^17^ implements ensemble algorithms to detect botnets in IoT networks. The research uses Random Forest and Gradient Boosted Decision Tree (GBDT) with Apache Spark tools to analyze the network traffic. Research^18^ integrates an optimized LightGBM classifier and Naive Bayes classifier in an IDS to detect botnet attacks in an IoT environment. Research^19^ uses various machine learning algorithms, such as artificial neural networks (ANN), decision trees (DT), Gaussian mixture models (GMM), and hierarchical clustering (HC), and then ensembles each pair of them. Research^20^ proposes botnet attack detection using three ensemble techniques: AdaBoosted, RUSBoosted, and bagged, with DT as the base machine learning algorithm.

In IoT networks, the number of connected devices is often dynamic and heterogeneous. The dynamics refer to the fluctuating nature of device connections, as new devices can join the network, and existing ones may disconnect. Heterogeneity pertains to the diverse types and functionalities of IoT devices, which can vary significantly in terms of capabilities, communication protocols, and purposes^21^. This dynamic and heterogeneous nature poses challenges for managing and maintaining IoT networks, as they need to be adaptable to changes in device connectivity and diverse device characteristics^22^.

The challenges become particularly apparent when considering an IDS that relies on a collective learning process involving all devices. In such cases, the IDS system might need to consider how the new data from the joining device influences the existing model and whether retraining is necessary. If the characteristics of the new device significantly differ from those of the existing devices in the dataset, it might be beneficial to restructure the dataset and retrain the model. This ensures that the IDS is well-equipped to handle the diverse nature of devices in the network^23^. We need approaches that can efficiently update the model with new data without discarding the existing knowledge and restructuring the dataset. This allows the IDS to adapt to changes in the network without the need for extensive restructuring and retraining. Given that problem, research^17–19^, cannot be applied to the issue at hand because they employ combined traffic from each device in the N-baIoT dataset to validate the proposed IDS model.

One of the studies attempts to propose two innovative approaches for feature extraction and classification, namely Logistic Regression (LR) and Artificial Neural Network (ANN). The evaluation process involves six devices from the N-BaIoT dataset. Notably, this research adopts an approach where each device’s traffic is analyzed individually, rather than combining all traffic into a single dataset. Consequently, machine learning models are generated separately for each device, utilizing specific datasets derived from the traffic generated by individual devices^24^. This research inspires us to help answer the problem of managing dynamic and heterogeneous devices of IoT that join the network.

In addressing the challenges posed by the dynamic and heterogeneous nature of IoT networks, this research advocates for approaches that facilitate the efficient updating of intrusion detection models with new data, ensuring adaptability without extensive restructuring. The research distinguishes itself by adopting a unique strategy-utilizing individual DNN training models for each IoT device. This allows for a detailed analysis of the characteristics of each device’s traffic without amalgamating all traffic data. Such an approach aligns with the need for adaptability in the face of changing network dynamics, enabling the IDS to comprehend and adjust to the distinct features of each device while preserving existing knowledge and model structures.

The concept is inspired by the incremental learning paradigm. Incremental learning encompasses the iterative process of acquiring fresh information or mastering additional skills while retaining previously acquired knowledge. This continuous learning approach involves the gradual enhancement and expansion of an existing model or system over time, diverging from the conventional practice of commencing anew with each new task or dataset. The essence of incremental learning lies in its adaptability and the ability to build upon existing knowledge, making it a valuable paradigm for scenarios where systems need to evolve and incorporate new insights without discarding the wisdom gained from prior experiences^25^.

The contributions and differences of this study compared to other research are as follows:

- This research proposes an ensemble NIDS anomaly detection model for botnet attack detection in IoT environments with heterogeneous IoT devices.
- The ensemble anomaly detection in this research uses a combination of ensemble averaging and DNN.
- The proposed model was tested using the N-BaIoT dataset, which contains botnet attacks in heterogeneous IoT devices.
- This research does not only analyze the accuracy, but we also consider other parameters such as precision, recall, F1-score, training time, and size of the training model.
- This research approach creates a DNN training model from individual devices and then averages the results from each of these individual devices. This strategy is employed to address the dynamic nature of the number of IoT devices connected to the IoT network. The model update process involves incorporating new data without discarding existing knowledge or restructuring the dataset.

## Methodology

**Figure 3:**
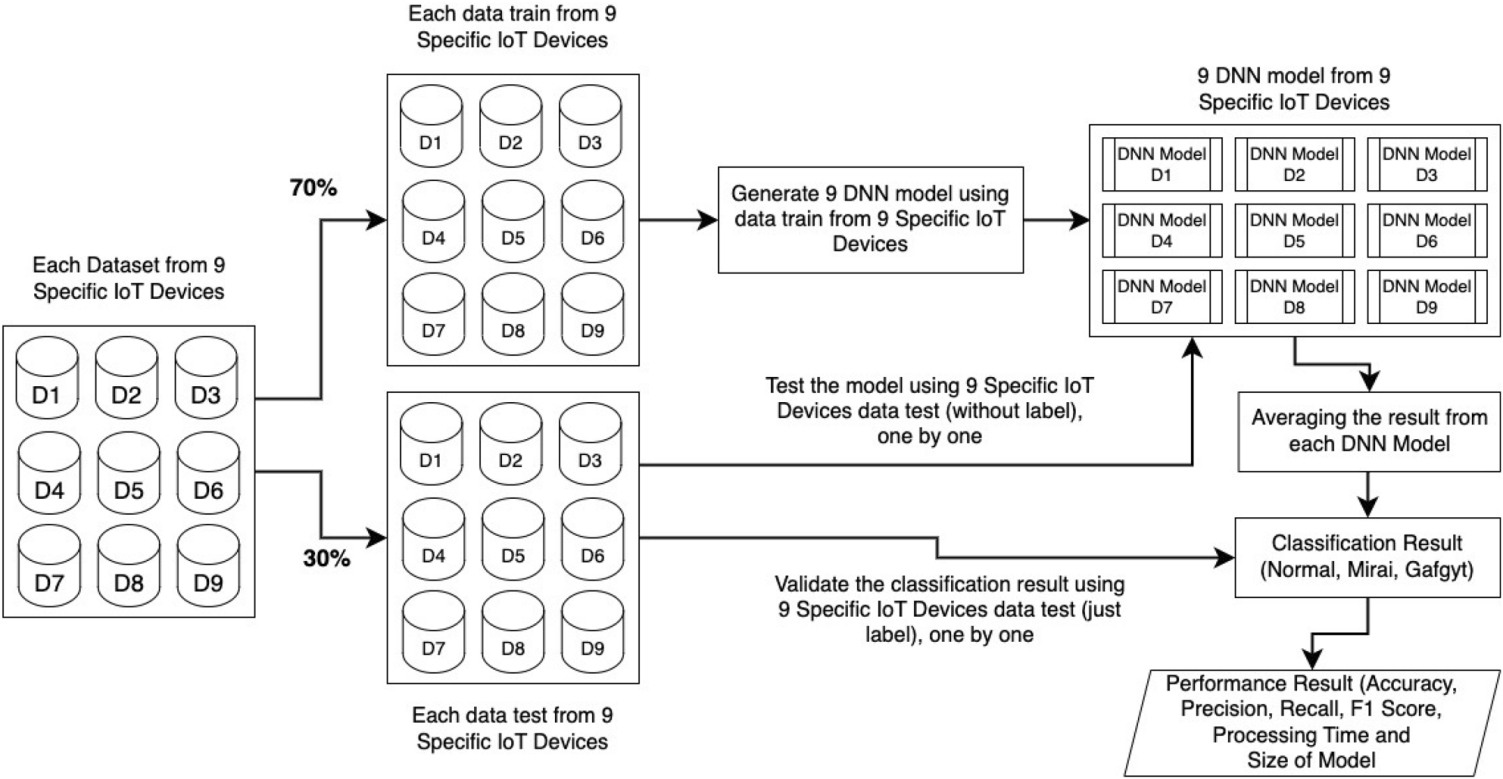
Classification process in our simulation approach.

The process diagram from this research can be seen in Fig. 3. The presented diagram encapsulates a systematic and structured approach to the development and testing of a machine learning model, specifically a DNN, employing data derived from distinct IoT devices. The stepwise process commences with the collection of datasets from nine specific IoT devices, setting the foundation for subsequent model development. This collected data is then intelligently divided, with 70% allocated for training the DNN model and the remaining 30% reserved for rigorous testing. The training phase involves the generation of nine unique DNN models, each attuned to the nuances of the data from the specified IoT devices.

Following the model generation, the testing phase ensues, where the reserved dataset is employed to evaluate the performance of each DNN model. This evaluation involves comparing model predictions against true values to gauge accuracy. Moreover, the validation step employs a separate dataset from the IoT devices to ensure the reliability and accuracy of each individual model. A notable aspect of this approach is the incorporation of an ensemble method, where the outputs from the nine DNN models are averaged for each test data point. This ensemble averaging contributes to determining the overall classification result, categorized as normal, Mirai, or Gafgyt indicating the system’s potential in anomaly detection or categorization of botnets in IoT devices.

The concluding phases involve a comprehensive evaluation of the system’s performance using diverse metrics, including accuracy, precision, recall, F1 score, processing time, and the size of the model. This meticulous evaluation ensures a thorough assessment of the effectiveness of the machine learning system in the context of IoT devices. Overall, the diagram provides a clear and visual representation of the entire process, emphasizing both the structural aspects of the model development and the critical performance metrics used for evaluation.

### Dataset preprocessing

The experiment started with preprocessing the N-baIoT dataset, which was obtained from research^10^. The dataset used in this research was a labeled version of the N-baIoT dataset and was prepared for the machine learning process. The dataset contains Mirai and BASHLITE botnet malware and attacks 9 different types of IoT devices, as shown in Table 1.

**Table 1:** Detail information of N-baIoT dataset.

ID	Device model	Device type	Number of benign	Mirai	BASHLITE
D1	Danmini	Doorbell	49548	Yes	Yes
D2	Ennio	Doorbell	39100	No	Yes
D3	Ecobee	Thermostat	13113	Yes	Yes
D4	Philips B120N/10	Baby monitor	175240	Yes	Yes
D5	Provision PT-737E	Security camera	62154	Yes	Yes
D6	Provision PT-838	Security camera	98514	Yes	Yes
D7	SimpleHome XCS7-1002-WHT	Security camera	46585	Yes	Yes
D8	SimpleHome XCS7-1003-WHT	Security camera	19528	Yes	Yes
D9	Samsung SNH 1011 N	Webcam	52150	No	Yes

**Table 2:** Mirai botnet attack activity in N-baIoT dataset.

Activity	Details
SCAN	Scanning process to find the vulnerability in the network
JUNK	Process to sending data for spamming
UDP	Flooding the UDP port
TCP	Flooding the TCP port
COMBO	Process to sending data for spamming with connect to specific IP address and port

**Table 3:** BASHLITE botnet attack activity in N-baIoT dataset.

Activity	Details
SCAN	Scanning process to find the vulnerability in the network
ACK	Flooding using ACK packet
SYN	Flooding using SYN packet
UDP	Flooding the UDP port
UDPPLAIN	Process flooding UDP using some options with packets per second in higher optimization

The dataset contains various labels from the botnet activity, such as Table 2 for Mirai botnet and Table 3 for BASHLITE botnet. In this research, we simplify the labels into “mirai” for Mirai botnet attack and “gafgyt” for BASHLITE botnet attack. Normal traffic is labeled with “BENIGN” label. Each dataset from each device is split into two data: 70% of the data is used for the training data and 30% for the testing data.

The dataset includes distinctive headers representing various stream aggregation attributes, each providing valuable insights into the characteristics of packet streams within the N-baIoT dataset. These attributes encompass H, which stands for “Source IP” in the N-BaIoT dataset, offering statistics summarizing recent traffic originating from the packet’s host based on IP. MI, denoting “Source MAC-IP” in the N-BaIoT dataset, provides stats summarizing recent traffic based on both IP and MAC addresses. HH, associated with “Channel” in the N-BaIoT dataset, involves statistics summarizing recent traffic from the packet’s host (IP) to the destination host. HH_jit, corresponding to “Channel jitter” in the N-BaIoT dataset, captures stats characterizing the jitter of traffic from the packet’s host (IP) to the destination host. Lastly, HpHp, signifying “Socket” in the N-BaIoT dataset, involves statistics summarizing recent traffic from the packet’s host and port (IP) to the destination host and port, exemplified by connections like 192.168.4.2:1242 -> 192.168.4.12:80. These stream aggregation attributes collectively contribute to a nuanced understanding of the packet stream dynamics and patterns within the N-baIoT dataset.

This research utilized all features from the dataset and did not employ a feature selection process. Instead, deep learning models were utilized, designed to effectively handle high-dimensional data and autonomously learn relevant features during the training process^26^. Feature selection algorithms can be computationally expensive, particularly when dealing with large datasets and a substantial number of features. In situations where computational resources are limited, the expenses associated with feature selection may surpass the potential benefits^27^. Given that this research focuses on IoT, a limitation arises regarding computational resources. In such scenarios, the simplicity and interpretability of the model may take precedence over achieving the highest predictive performance. Consequently, opting to use all available features might be considered more favorable.

### NIDS model

**Figure 4:**
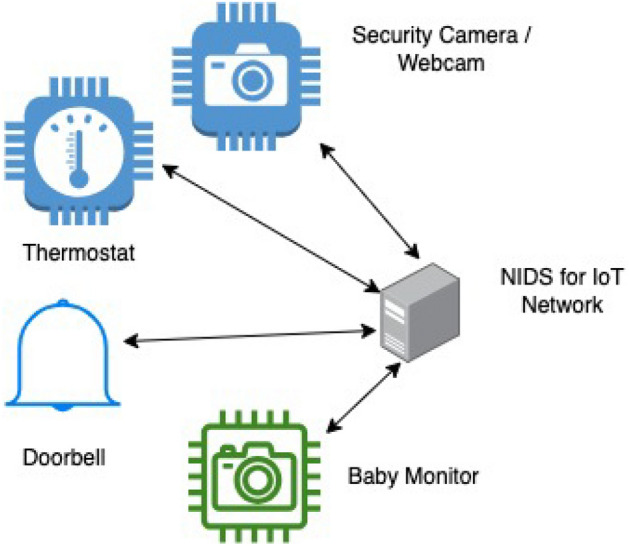
Our view in NIDS architecture for IoT network with heterogeneous IoT device.

Our proposed Network Intrusion Detection System (NIDS) architecture for the Internet of Things (IoT) environment, as illustrated in Fig. 4, envisions a model tailored to the intricacies of a diverse range of IoT devices. In our NIDS model, traffic emanating from each IoT device becomes instrumental in generating a device-specific training model through the utilization of a Deep Neural Network (DNN). This training model, unique to each device, is subsequently employed to predict network traffic and identify potential anomalies. The predictions from individual models are then aggregated using the ensemble averaging method, culminating in a final result that leverages the collaborative insights from multiple devices.

This collaborative Intrusion Detection System (IDS) model is designed for implementation within a centralized framework, such as a collaborative IDS hosted on a central server or a fog device in real-world deployment scenarios. Notably, in our simulation environment for this research, we emulate the centralized implementation, envisioning the real-world execution where all processes are consolidated in a central server or fog device. This simulation environment allows for a comprehensive evaluation of the proposed NIDS architecture’s efficacy, offering insights into its performance under controlled conditions. The envisioned deployment in a centralized collaborative IDS aligns with the increasing trend in IoT security solutions, providing a centralized and efficient approach to detect and mitigate potential threats across a diverse array of IoT devices.

### Ensemble averaging DNN

This research proposes an ensemble averaging DNN by ensembling each DNN training model from every heterogeneous IoT device. Each device in IoT has its own characteristics, so generating individual training models from each device is expected to capture the characteristics of each heterogeneous device in the IoT environment. This research creates 9 DNN training models using 70% of the traffic from each device in the N-BaIoT dataset (Table 1). After that, the prediction results from each training model are averaged using the ensemble averaging method. This research uses 30% of the traffic from each device in the N-BaIoT dataset (Table 1) to test the proposed mechanism.

**Algorithm 1:**
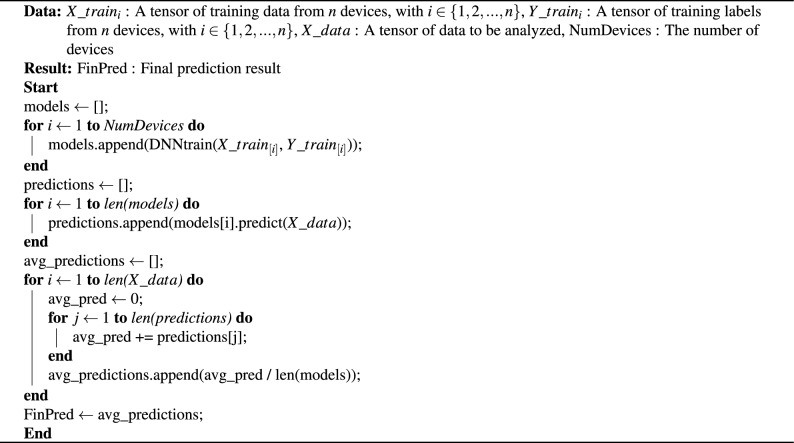
Ensemble Averaging DNN

The detailed ensemble averaging DNN mechanism for botnet attack detection in heterogeneous devices can be seen in Algorithm 1. The algorithm described here, named “Ensemble Averaging DNN,” is designed to harness the collective capabilities of multiple devices to enhance the predictive performance of a Deep Neural Network (DNN) model. The input to the algorithm includes training data ($X\_train_{i}, Y\_train_{i}$) from $n$ devices, a tensor $X\_data$ representing data to be analyzed, and the number of devices ($NumDevices$). The process begins by initializing an empty list called models to store the trained DNN models. Subsequently, the algorithm iterates over each device, trains an individual DNN model, and appends it to the models list. After training, the algorithm proceeds to generate predictions. It initializes an empty list called predictions to store predictions made by each individual model. The algorithm then iterates over each model in the models list, predicting the output for the input data $X\_data$ and appending each prediction to the predictions list.

Following the generation of predictions, the algorithm enters a phase of averaging predictions. It initializes an empty list called avg_predictions to store averaged predictions. The algorithm iterates over each element in the input data $X\_data$ and, for each element, iterates over each prediction in the predictions list. It sums up the predictions and calculates the average prediction for the current element by dividing the sum by the total number of models. The averaged prediction is then appended to the avg_predictions list. Finally, the algorithm assigns the avg_predictions list to the variable FinPred, representing the final ensemble-averaged prediction. In essence, this algorithm aims to collaboratively train multiple DNN models on diverse data sources, generate individual predictions, and then aggregate these predictions through averaging to produce a robust and generalized final prediction.

**Table 4:** DNN hyperparameter.

Hyperparameter	Details
Input dimension	115
Hidden layer	4
Output dimension	3 (multi-class)
Output layer activation function	Softmax
Optimisation algorithm	Adam
Batch size	1000
Epochs	10
Loss function	Categorical crossentropy
Metrics	Accuracy, recall, precision, F1-score

**Table 5:** Hidden layer details.

Hidden layer	Number of node	Activation function
1	13	ReLU
2	11	ReLU
3	9	ReLU
4	7	ReLU

The detailed information of the hyperparameters from the DNN used in this research can be seen in Table 4. In Table 4, the input dimension is specified as 115 features, with a neural network structure comprising 4 hidden layers and an output layer designed for multi-class classification with 3 classes. The output layer employs the Softmax activation function, and the optimization algorithm chosen for training is Adam. The batch size for training is set to 1000, with 10 epochs used to iterate through the entire training dataset. The loss function employed is Categorical Crossentropy, and evaluation metrics include Accuracy, Recall, Precision, and F1-Score.

**Figure 5:**
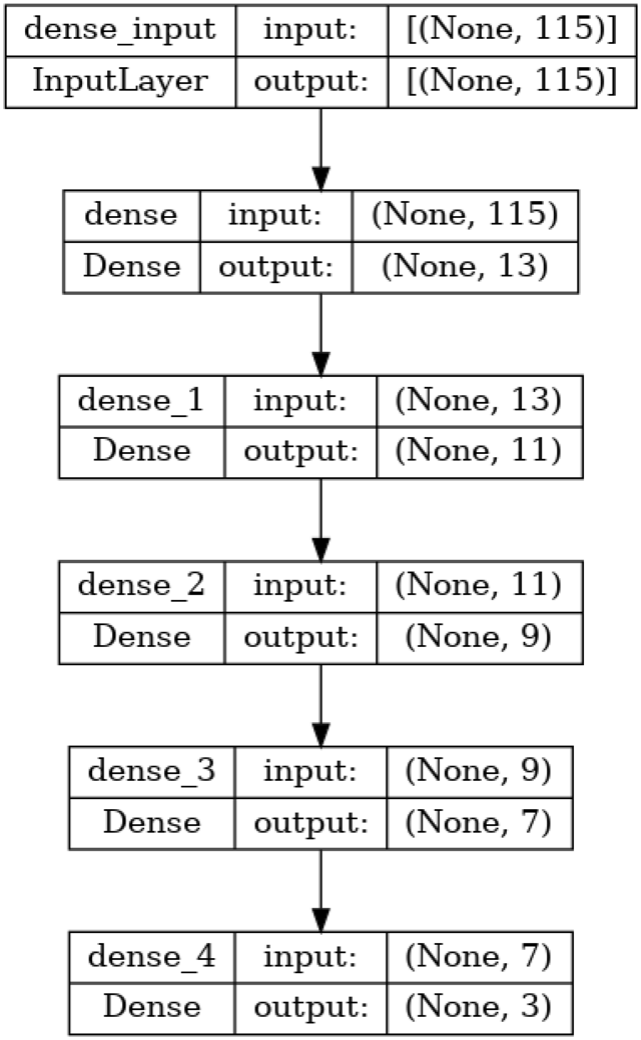
DNN Architecture for each devices.

Table 5 provides insights into the specifics of each hidden layer within the neural network architecture. The hidden layers are indexed from 1 to 4, and the number of nodes in each layer decreases progressively from 13 to 7. The activation function applied to the nodes in each hidden layer is Rectified Linear Unit (ReLU), a common choice for introducing non-linearity in neural networks. Collectively, these tables furnish a comprehensive overview of the DNN’s configuration, facilitating a clear understanding of the model’s structure and training settings for the specified machine-learning task. The detailed visualization of DNN architecture from Tables 4 and 5 can be seen in Fig. 5.

### Performance parameter

In this research, some parameters were used to evaluate the benchmarking study. The accuracy, F1-score, precision, and recall of the algorithms were measured based on the benchmarking scenarios. These are all important evaluation metrics for classification tasks because they provide different perspectives on the performance of a classification model. Each metric offers insights into different aspects of the model’s predictive capabilities and can help assess its effectiveness in solving the classification problem.

Additionally, the size of the training model produced by each algorithm and the time taken for the training and testing processes were also measured in this research. These metrics play important roles in the practical deployment and performance of classification models. They influence resource utilization, cost, latency, scalability, and user experience. Balancing model size, training time, and prediction time is crucial to ensure efficient, effective, and practical solutions for classification tasks in real-world scenarios.

1. Accuracy (%). The accuracy of an IDS is critical to ensure that it can effectively identify and respond to malicious activity while minimizing the number of false positives. High accuracy means the system correctly identifies true positives and true negatives. The accuracy is calculated using Eq. (6).
  6 $$\begin{aligned} \frac{TP+TN}{TP+FP+TN+FN} = Accuracy \end{aligned}$$
  Where *TP* is true positive, TN is true negative, FP is false positive, and FN is false negative. Precision (%): The precision in IDS is the ratio of true positive predictions to the total number of positive predictions made by the model. It measures the accuracy of positive predictions. Specifically, the proportion of correctly predicted positive instances out of all instances predicted as positive. The precision parameter is calculated using Eq. (7).
  7 $$\begin{aligned} \frac{TP}{TP+FP} = Precision \end{aligned}$$
2. Recall (%): The recall metric, also known as sensitivity or true positive rate, is the ratio of true positive predictions to the total number of actual positive instances in the dataset. It measures the model’s ability to correctly identify positive instances, regardless of whether some instances are incorrectly predicted as negative. The recall parameter is calculated using Eq. (8).
  8 $$\begin{aligned} \frac{TP}{TP+FN} = Recall \end{aligned}$$
3. F1-Score (%): The F1 score is a widely used evaluation metric in machine learning, particularly for classification tasks. It is a measure of the model’s accuracy that combines both precision and recall into a single value. The F1 score provides a balanced assessment of the model’s performance, especially in situations where false positives and false negatives carry similar importance. The F1 score is calculated using Eq. (9).
  9 $$\begin{aligned} \frac{2*(Precision*Recall)}{Precision + Recall} = F1-Score \end{aligned}$$
4. Processing Time (seconds (s)): Processing time consists of training time and prediction time in machine learning. Training time refers to the time it takes for a model to learn from a given dataset and adjust its parameters to optimize its performance. Prediction time refers to the time it takes for the trained model to make predictions on new unseen data. The processing time has been defined by Eq. (10).
  10 $$\begin{aligned} TT + PdT = PcT \end{aligned}$$
  Where *TT* is Training Time, *PdT* is Prediction Time, and *PcT* is Processing Time.
5. Size of Training Model (kilobyte (Kb)). Each machine learning or ensemble learning training process will produce a training model. All training models can be saved into storage and used again for the prediction process in anomaly detection. The larger the training model, the more storage capacity is needed to store it. Therefore, measuring the size of the training model as a benchmarking parameter is essential for analyzing performance.

**Table 6:** Benchmarking scenario term list.

ID	Details	ID	Details	ID	Details
TR1	Training data from device ID D1	M1	Training model from TR1	TS1	Testing data from device ID D1
TR2	Training data from device ID D2	M2	Training model from TR2	TS1	Testing data from device ID D1
TR3	Training data from device ID D3	M3	Training model from TR3	TS3	Testing data from device ID D3
TR4	Training data from device ID D4	M4	Training model from TR4	TS4	Testing data from device ID D4
TR5	Training data from device ID D5	M5	Training model from TR5	TS5	Testing data from device ID D5
TR6	Training data from device ID D6	M6	Training model from TR6	TS6	Testing data from device ID D6
TR7	Training data from device ID D7	M7	Training model from TR7	TS7	Testing data from device ID D7
TR8	Training data from device ID D8	M8	Training model from TR8	TS8	Testing data from device ID D8
TR9	Training data from device ID D9	M9	Training model from TR9	TS9	Testing data from device ID D9
ENS	Ensemble training model from M1 - M9				

### Benchmarking scenario

This research has two categories of benchmarking scenarios: preliminary analysis and proposed model result. The preliminary analysis section consists of Scenario 1 and Scenario 2. The preliminary analysis section was used to answer research questions 1 and 2. The proposed model result section consists of scenario 3. The proposed model result is used to answer research question 3. There are some term lists to make it easier to explain benchmarking scenario results in the result and discussion section. Table 6 shows the term list from the benchmarking scenario. The detail of each scenario is below:

1. Scenario 1: The first scenario involves benchmarking the performance of each DNN training model from every device (M1–M9) and testing using testing data from their device traffic (TS1–TS9). So, M1 will test using TS1, M2 will test using TS2, and so on. This scenario aims to assess the performance of the training model from each device using their data test from each device traffic. It is designed to answer research question 1.
2. Scenario 2: The second scenario aims to evaluate the performance of each DNN training model from every device (M1–M9) and cross-test using testing data from each other device (TS1–TS9). So, M1 will test using M2–M9 traffic, M2 will test using M1 and M3–M9 traffic, and so on. This scenario aims to see the performance of the training model in each device when analyzing the traffic from another device. This scenario is designed to answer research question 2.
3. Scenario 3: The third scenario benchmarks the performance of the proposed ensemble averaging DNN (ENS) and tests using each device’s traffic (TS1–TS9). This scenario is designed to answer research question 3.

## Result and discussion

In this section, the results of the simulation modeling and benchmarking study are presented and discussed. The findings of this research are discussed in the context of their impact on ensemble averaging for NIDS in heterogeneous IoT devices. Additionally, potential areas for future research in this field are highlighted.

### Experiment environment

This research used a server with the following specifications: Processor 2.3 GHz 16-Core Intel(R) Xeon(R) CPU E5-2650 v3 and 128 GB memory. The operating system used was Ubuntu 22.04.2 LTS. Python version 3.10.6 and Keras version 2.12 were employed as the machine learning library for conducting the DNN experiments. Jupyter notebook version 6.5.3 was used for presenting the experiment and simulation results.

### Preliminaries analysis

In this section, the explanation of results from both Scenario 1 and Scenario 2 is provided. The main objective of Scenario 1 was to assess the performance of individual DNN models constructed using device-specific traffic for the purpose of detecting botnet attacks occurring within the traffic of each respective device.

**Table 7:** Scenario 1 result.

Model	Testing data	Accuracy	Precision	Recall	F1-score	Training time (s)	Prediction time (s)	Processing time (s)	Size of model (Kb)
M1	TS1	100	99.9	100	100	31	16	47	70.3
M2	TS2	100	99.9	100	99.9	11	5	16	70.3
M3	TS3	100	99.9	99.9	99.9	21	12	33	70.4
M4	TS4	100	100	100	100	31	16	47	70.4
M5	TS5	100	99.9	99.9	99.9	21	14	35	70.4
M6	TS6	100	99.9	99.9	99.9	21	13	34	70.4
M7	TS7	100	99.9	99.9	99.9	21	12	33	70.4
M8	TS8	99.9	99.6	99.8	99.7	22	12	34	70.4
M9	TS9	100	100	100	100	11	5	16	70.4

The results of Scenario 1 are presented in Table 7. The findings indicate that the DNN models within each device exhibited robust performance when analyzing the traffic generated by that specific device. Notably, accuracy for each device reached 100%, signifying accurate identification of both true positive and true negative instances of botnet attacks within the corresponding device’s traffic. Precision and recall metrics also demonstrated performance exceeding 99%, implying the models’ ability to minimize misclassifications of normal traffic while accurately recognizing positive instances. Moreover, the DNN models achieved a high F1-score in detecting botnet attacks, highlighting their proficiency in both precision and recall aspects. Both training and prediction times for each model were influenced by dataset volume, with larger datasets leading to longer training and prediction durations. Remarkably, the model size remained consistent at around 70 Kb for each DNN model, indicating a stable size unaffected by variations in training data volume.

**Figure 6:**
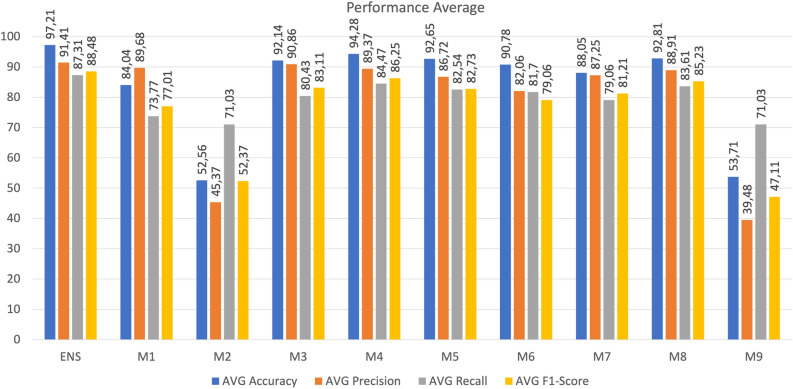
Average accuracy, recall, precision, and F1-score from ensemble averaging DNN when compared with single device DNN in analyzing traffic on each device.

Scenario 2 aimed to evaluate the performance of individual DNN models constructed using device-specific traffic to detect botnet attacks within traffic from other devices. In this scenario, each DNN model developed from one device’s traffic was applied to analyze testing data from different devices. Results from Scenario 2 are documented in Table 8. The outcomes revealed that performance metrics (accuracy, precision, recall, and F1-score) of DNN models when analyzing testing data from different devices were notably lower compared to their performance when analyzing data from their own device’s traffic. This suggests that training models from each device were optimized for analyzing traffic originating from their respective devices and demonstrated reduced effectiveness when applied to traffic analysis from different devices.

**Table 8:** Scenario 2 result.

Model	Testing data	Accuracy	Precision	Recall	F1-score	Training time (s)	Prediction time (s)	Processing time (s)	Size of model (Kb)
M1	TS1	100	99.9	100	100	31	16	47	70.3
	TS2	8.4	60	9.4	15.9	31	5	36	70.3
	TS3	95.4	97	87.2	91.3	31	12	43	70.3
	TS4	99.2	99.2	98.4	98.7	31	18	49	70.3
	TS5	97.8	98.3	92.7	95.1	31	10	41	70.3
	TS6	96.5	97.2	91.7	94	31	10	41	70.3
	TS7	92.7	94.2	80.1	84.9	31	13	44	70.3
	TS8	94.2	95.8	75.6	81.1	31	10	41	70.3
	TS9	72.2	65.6	28.9	32.1	31	5	36	70.3
M2	TS1	16.2	11.3	45.4	17.6	11	14	25	70.3
	TS2	100	99.9	100	99.9	11	5	16	70.3
	TS3	38.4	22.5	66	33.4	11	14	25	70.3
	TS4	44	44	66	49.6	11	14	25	70.3
	TS5	47	35.2	65.5	45.5	11	12	23	70.3
	TS6	48.4	40	66.1	48.9	11	12	23	70.3
	TS7	40.1	32.9	65.3	43.2	11	11	22	70.3
	TS8	39.2	22.8	65.6	33.7	11	12	23	70.3
	TS9	99.8	99.8	99.4	99.6	11	6	17	70.3
M3	TS1	97.8	98	93.7	95.7	21	16	37	70.4
	TS2	59.7	64.6	27.8	36.1	21	5	26	70.4
	TS3	100	99.9	99.9	99.9	21	12	33	70.4
	TS4	96.5	96.4	93.8	94.7	21	14	35	70.4
	TS5	97.6	98	93	95.2	21	13	34	70.4
	TS6	96.6	97.2	91.9	94	21	13	34	70.4
	TS7	98.2	98.4	90.1	93.4	21	12	33	70.4
	TS8	99.8	99.4	97.4	98.4	21	12	33	70.4
	TS9	83.1	65.9	36.3	40.6	21	5	26	70.4
M4	TS1	99.4	99	99.4	99.2	31	16	47	70.4
	TS2	79.4	61.8	56.8	58.8	31	13	44	70.4
	TS3	95.9	95.5	96.5	95.9	31	5	36	70.4
	TS4	100	100	100	100	31	16	47	70.4
	TS5	99.4	99.3	99.2	99.3	31	19	50	70.4
	TS6	98.9	99.1	98.3	98.7	31	14	45	70.4
	TS7	94.5	94.5	83.5	87.5	31	6	37	70.4
	TS8	95	88.8	78.5	82.1	31	13	44	70.4
	TS9	86.1	66.4	48.1	54.8	31	14	45	70.4
M5	TS1	95.7	94.4	97.5	95.8	21	16	37	70.4
	TS2	85.5	62.6	37	41.2	21	6	27	70.4
	TS3	88.8	82.8	89.2	85.6	21	13	34	70.4
	TS4	99.4	99.4	98.8	99.9	21	18	39	70.4
	TS5	100	99.9	99.9	99.9	21	14	35	70.4
	TS6	99.9	99.9	99.8	99.9	21	13	34	70.4
	TS7	89.5	89.4	91	90.2	21	13	34	70.4
	TS8	89.3	85.9	90.7	88.1	21	14	35	70.4
	TS9	85.8	66.2	39	44	21	6	27	70.4
M6	TS1	68.8	56.1	72.2	54.4	21	13	34	70.4
	TS2	89.3	64.8	44.5	50.4	21	5	26	70,4
	TS3	92.6	80.6	94	85.5	21	12	33	70.4
	TS4	98.5	98.3	98.6	98.5	21	16	37	70.4
	TS5	99.8	99.8	99.8	99.8	21	11	32	70.4
	TS6	100	99.9	99.9	99.9	21	13	34	70.4
	TS7	92.6	89.4	93.7	91.3	21	11	32	70.4
	TS8	92.4	83.8	94.3	88	21	15	36	70.4
	TS9	83.1	65.9	38.3	43.8	21	4	25	70.4
M7	TS1	99	98.8	99	98.9	21	14	35	70.4
	TS2	26.9	53.6	19.9	28.4	21	6	27	70.4
	TS3	99.8	99.3	97.1	98.1	21	13	34	70.4
	TS4	95.7	95.6	93.7	94.3	21	13	34	70.4
	TS5	95.1	96.2	84.8	88.7	21	12	33	70.4
	TS6	92.4	94.1	83.2	86.5	21	13	34	70.4
	TS7	100	99.9	99.9	99.9	21	12	33	70.4
	TS8	99.9	99.5	99.6	99.5	21	11	32	70.4
	TS9	83.7	48.3	34.4	36.6	21	4	25	70.4
M8	TS1	98.4	98.3	91.8	94.5	22	16	38	70.4
	TS2	61.6	44.3	32.4	37.2	22	5	27	70.4
	TS3	99.9	99.2	99.8	99.5	22	9	31	70.4
	TS4	98.7	98.5	98.3	98.4	22	16	38	70,4
	TS5	99.1	98.2	98.1	98.2	22	10	32	70.4
	TS6	99.3	98.9	98.6	98.8	22	12	34	70.4
	TS7	99.4	99.1	97.2	98.1	22	13	35	70.4
	TS8	99.9	99.6	99.8	99.7	22	12	34	70.4
	TS9	79	64.1	36.5	42.7	22	4	26	70.4
M9	TS1	35.8	20.8	66.2	31.3	11	16	27	70.4
	TS2	97	89.7	97.7	93.2	11	4	15	70.4
	TS3	38.7	18	66.4	25.9	11	11	22	70.4
	TS4	39.5	29.9	56.5	37.8	11	13	24	70.4
	TS5	45.7	25.5	59.3	35.2	11	12	23	70.4
	TS6	47.2	29.6	62.6	40.1	11	10	21	70.4
	TS7	40.1	22.6	64.4	32.8	11	12	23	70.4
	TS8	39.4	19.3	66.2	27.7	11	13	24	70.4
	TS9	100	100	100	100	11	5	16	70.4

In summary. the evaluation of DNN model performance highlighted their effectiveness in analyzing traffic from their respective devices. However, it also revealed reduced performance when confronted with traffic from diverse devices. This emphasizes the importance of training models on data that accurately represents the specific environment, ensuring optimal results.

### Proposed model result

The preliminary analysis results indicate that the DNN model from each device is unable to analyze testing data from other devices. This implies that the traffic characteristics in each device differ, reflecting the heterogeneity of devices within the IoT environment. An IDS with a comprehensive perspective is needed to analyze botnet attacks across these diverse devices in the IoT environment. To address this, the research proposes an ensemble averaging approach using DNN to establish a unified view for analyzing botnet attacks across heterogeneous devices within the IoT environment.

**Table 9:** Scenario 3 result.

Model	Testing data	Accuracy	Precision	Recall	F1-score	Training time (s)	Prediction time (s)	Processing time (s)	Size of model (Kb)
ENS	TS1	99.5	99.3	99.6	99.5	190	132	322	633.4
	TS2	89	61.6	45.8	51.3	190	41	231	633.4
	TS3	99.2	99.1	99.5	99.3	190	108	298	633.4
	TS4	99.1	99	99.2	99.1	190	129	319	633.4
	TS5	99.9	99.8	99.8	99.8	190	111	301	633.4
	TS6	99.9	99.9	99.8	99.9	190	110	300	633.4
	TS7	99	98.7	98.6	98.7	190	114	304	633.4
	TS8	98.8	98.7	99.2	98.9	190	111	301	633.4
	TS9	90.5	66.6	44.3	49.9	190	48	238	633.4

The objective of the third scenario is to assess the performance of the ensemble averaging DNN model in detecting botnet attacks within the traffic of each heterogeneous device. The results of Scenario 3 are presented in Table 9. The proposed model demonstrates a performance level exceeding 89% accuracy in detecting botnet attacks within IoT environments characterized by heterogeneous devices. Additionally, the model achieves precision, recall, and F1-score metrics surpassing 98% when analyzing botnet attacks across most heterogeneous devices. However, exceptions occur for testing data from devices 2 and 9 due to device vulnerabilities specific to the BASHLITE botnet, resulting in the absence of Mirai botnet attacks. Consequently, this imbalance in traffic contributes to precision, recall, and F1-score falling below 98%.

To compare the performance of the ensemble averaging DNN with single DNN models from each device, the research averages the results from accuracy, precision, recall, and F1-score in Tables 8 and 9, as shown in Fig. 6. The findings reveal that the ensemble averaging DNN outperforms single DNN models from individual devices in terms of accuracy, precision, recall, and F1-score when detecting botnet attacks across the entire spectrum of IoT heterogeneous devices. This underscores the model’s ability to comprehensively consider all IoT heterogeneous devices, accurately identifying true positives and negatives, minimizing incorrect classifications of normal traffic, and correctly recognizing positive instances.

The training time for the ensemble averaging DNN is longer than that of individual models from each device due to its utilization of all individual training models from heterogeneous devices. Thus, training time is contingent on the number of devices and the training time required for generating individual models. In this research, the training time encompasses the total time spent training the DNN model constructed from traffic data of nine distinct IoT devices. Prediction time for the ensemble averaging DNN is similarly dependent on the total number of training models. Each testing data point is predicted using every training model, and the prediction outcomes are averaged through an ensemble averaging process. In this research, prediction time accounts for the total prediction instances using the nine DNN training models. The model size of the ensemble averaging DNN comprises the cumulative size of all nine training models.

### Computational complexity analysis

The computational complexity of the “Ensemble Averaging DNN” algorithm (Algorithm 1) is a critical aspect to assess, considering its application in detecting botnet attacks across multiple heterogeneous IoT devices. The algorithm encompasses several phases, each contributing to the overall complexity. In the model training phase (Lines 3–5), the algorithm iterates over each of the NumDevices IoT devices, involving the training of individual DNN models. The training complexity for one DNN model is denoted as $O(T_{\text {DNN}})$, encapsulating processes such as forward and backward passes, weight updates, and iterative epochs. Consequently, the total training complexity for all devices becomes $O(\text {NumDevices} \times T_{\text {DNN}})$, emphasizing the computational burden associated with training diverse models for each IoT device.

Following model training, the prediction phase (Lines 7–8) involves generating predictions for the input data using each of the trained DNN models. The prediction complexity for one model is denoted as $O(P_{\text {DNN}})$, accounting for the intricacies of model prediction. The total prediction complexity for all models becomes $O(\text {len(models)} \times P_{\text {DNN}})$, underscoring the computational demands associated with predicting outcomes across multiple models. Subsequently, the algorithm proceeds to average predictions (Lines 9–15), introducing two nested loops that iterate over the input data and predictions. The complexity of this averaging step is $O(N \times M)$, where $N$ is the length of predictions and $M$ is the number of models. This phase adds an additional layer of complexity, reflecting the computational cost of aggregating predictions from diverse models.

**Figure 7:**
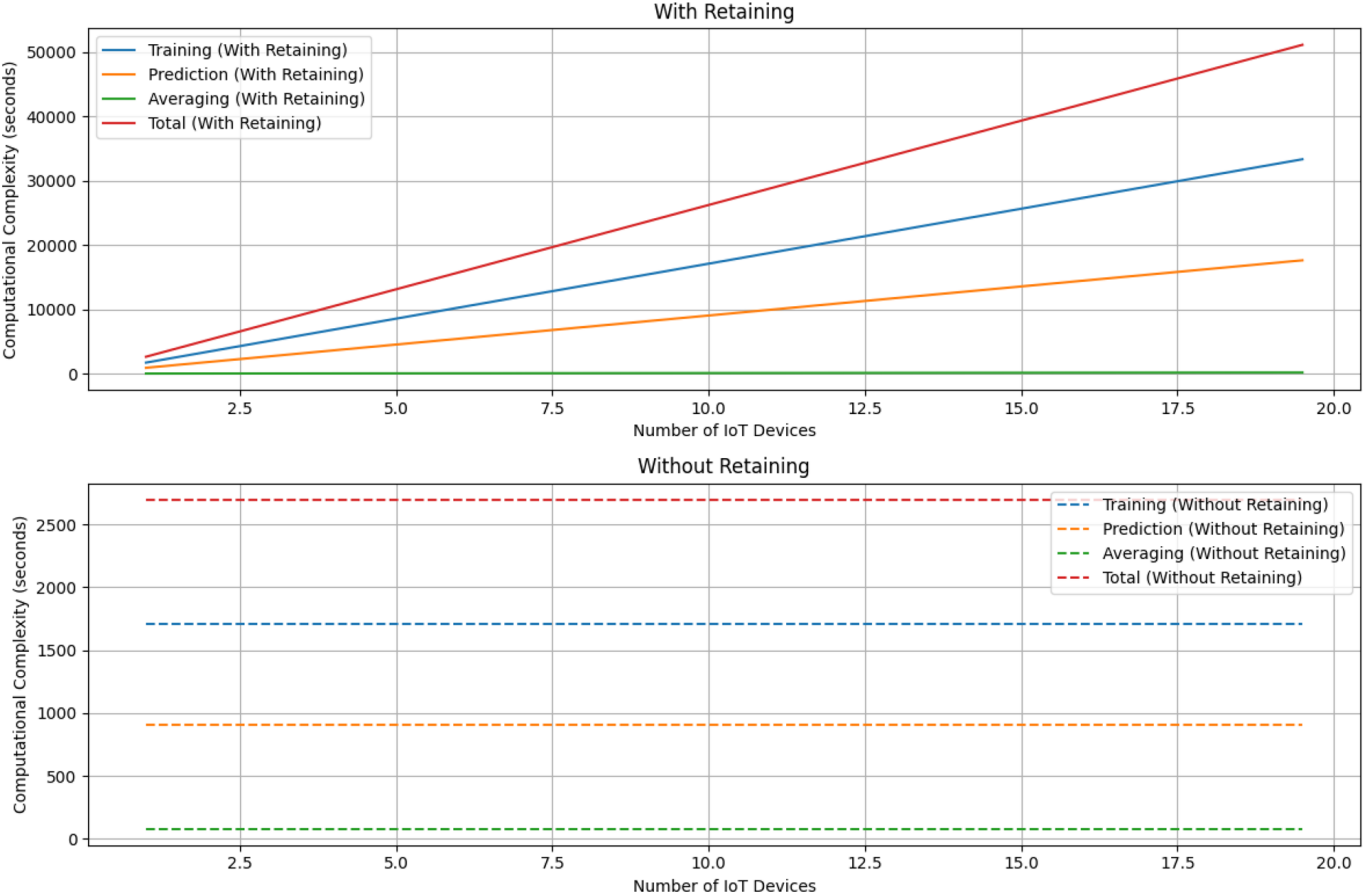
Computational complexity simulation.

In this section, we aim to simulate the computational complexity using the average trends of data obtained from the experimental section. The computational complexity of the algorithm involves three primary components: training, prediction, and averaging. The training complexity ($O(\text {NumDevices} \times T_{\text {DNN}})$) depends on the number of IoT devices ($\text {NumDevices}$) and the training time for one DNN model ($T_{\text {DNN}}$). It explores how the computational complexity varies with the number of IoT devices involved, examining both scenarios with and without retaining knowledge. In the context of the N-baIoT dataset with nine devices and the associated training times, the overall training complexity would be influenced by the cumulative training time for all devices. Similarly, the prediction complexity ($O(\text {len(models)} \times P_{\text {DNN}})$) is influenced by the number of models ($\text {len(models)}$) and the prediction time for one model ($P_{\text {DNN}}$). Given the diverse characteristics of the IoT devices and their associated prediction times, optimizing the prediction phase becomes crucial for efficiency. The averaging phase complexity ($O(N \times M)$) involves the length of predictions ($N$) and the number of models ($M$). In the given scenario, the length of predictions corresponds to the size of the testing dataset, and the number of models is nine (as there are nine IoT devices). This phase’s computational cost is influenced by the quantity of predictions and the number of models involved. Based on Table 9, the average training time for the ensemble averaging DNN model (in seconds) is 190, and the average prediction time for the ensemble averaging DNN model (in seconds) is 100.44.

The result of computational complexity can be seen in Fig. 7. The trend of the data in the graph shows that as the number of IoT devices increases, the computational complexity also increases. The training complexity exhibits a linear increase, indicating that the time required for training the system grows steadily with the number of devices. On the other hand, the prediction complexity increases at a much slower rate, suggesting that the time required for making predictions is less affected by the number of devices. The averaging complexity remains relatively constant, indicating that this aspect of computational complexity is not significantly impacted by the number of IoT devices. The total complexity, represented by the sum of the other complexities, grows nonlinearly, with the training complexity having the most significant influence on the overall computational complexity. This trend suggests that as the number of IoT devices increases, the training process becomes the dominant factor contributing to the total computational complexity.

The observed data trend in the graph illustrates a direct relationship between the number of IoT devices and computational complexity, indicating that as the count of devices rises, so does the overall computational load. However, it’s crucial to interpret this trend within the context of the incremental learning paradigm. The initial increase in complexity aligns with the conventional understanding that more devices lead to higher computational demands, especially during the initial phases of learning. Notably, as the learning process progresses and the model adapts to the existing devices, the graph suggests a potential stabilization or slower growth in complexity.

One distinctive advantage of incremental learning becomes apparent in this context. With the incorporation of a new device, the system doesn’t necessitate a complete reiteration of the training process. Instead, it intelligently builds upon the existing knowledge, leveraging prior learning experiences. This means that after the initial training phase, the impact of adding new devices on the overall computational complexity might not follow a linear trajectory. The system optimizes its efficiency by selectively updating its knowledge base, demonstrating the adaptability inherent in incremental learning. This nuanced perspective provides a more insightful understanding of the relationship between device count and computational complexity, showcasing the efficiency gains afforded by incremental learning in dynamically evolving IoT environments.

### Discussion

The research demonstrates the effectiveness of ensemble learning, specifically the ensemble averaging DNN approach, in addressing the challenges of detecting botnet attacks in diverse IoT devices. The study reveals that by employing ensemble learning, the algorithm creates subsets of training data, leveraging diverse behaviors among training models. This strategic utilization of diverse models contributes to enhanced prediction accuracy, as showcased by the average accuracy of 97.21, precision of 91.41, recall of 87.31, and an F1-score of 88.48. The research confirms that the collaborative nature of ensemble learning observed through the combination of individual DNN models, results in a robust intrusion detection strategy capable of handling the intricacies of IoT environments with heterogeneous devices. In essence, the study provides evidence that ensemble learning, as applied in the research, contributes to better predictions by utilizing the distinctions among training models, addressing the specific challenges of botnet attack detection in IoT networks.

The computational complexity analysis of the “Ensemble Averaging DNN” algorithm is crucial for its application in detecting botnet attacks across multiple heterogeneous IoT devices. The algorithm comprises phases like model training, prediction, and averaging, each contributing to overall complexity. The training phase involves iterating over NumDevices IoT devices, resulting in $O(\text {NumDevices} \times T_{\text {DNN}})$ complexity. Prediction complexity ($O(\text {len(models)} \times P_{\text {DNN}})$) accounts for the intricacies of model prediction. The averaging phase complexity ($O(N \times M)$) reflects the computational cost of aggregating predictions. Simulated computational complexity, influenced by training, prediction, and averaging, demonstrates an increase with the number of IoT devices. The training complexity exhibits linear growth, whereas prediction complexity increases at a slower rate. Averaging complexity remains constant, and the total complexity grows nonlinearly, emphasizing the training phase’s dominance. The observed trend aligns with expectations, but incremental learning mitigates the impact of adding new devices, optimizing efficiency and showcasing adaptability in evolving IoT environments.

Our single DNN approach in every device achieves 100% of accuracy, so the average of our DNN approach is to achieve 100% accuracy. If we compare with^17–19^ our single DNN approach can compete with the result from this research. When we talk about ensemble averaging results in our approach, the accuracy is not quite good. However, if we talk about the security of dynamic IoT devices and simple scenarios to update the model with new data without discarding the existing knowledge and restructuring the dataset, our approach is more feasible to implement than other research. Our approach can not be a direct head-to-head comparison because there is a difference in validating methods to overcome the formulated problem.

### Future works

This study concentrates on an ensemble approach employing DNN models, where prediction results are equally averaged to identify botnet attacks within IoT environments characterized by heterogeneous devices. However, a limitation of the proposed model arises from its inability to manage imbalanced data within individual devices. Certain devices may lack certain types of botnet attacks due to their inherent resistance, leading to imbalanced traffic data. Therefore, for future research, the incorporation of a balancing averaging method becomes essential.

Furthermore, there exists potential for extending this research towards the creation of a decentralized ensemble averaging framework. This could involve decentralizing the DNN training process across IoT devices. Subsequently, the resultant training model outcomes could be shared among devices to facilitate the ensemble prediction procedure. Technologies like peer-to-peer connections could be employed within each device to achieve this goal. Additionally, devices would need the capability to undertake DNN model training and prediction processes using multiple DNN models. It’s worth noting that this investigation employed a homogeneous IoT network with heterogeneous devices. Future research could explore the implementation of ensemble averaging within a heterogeneous network. This approach could lead to the development of collaborative Intrusion Detection Systems (IDS) aimed at detecting significant attacks across the entire network.

In reflecting on the outcomes of our study, it is essential to address potential limitations that may impact the generalizability and applicability of our proposed ensemble averaging DNN for detecting botnet attacks across heterogeneous IoT devices. Firstly, while our approach exhibits commendable performance in capturing the diverse traffic patterns across the IoT devices considered in our study, it is crucial to acknowledge that the presented results may be influenced by the specific characteristics of the devices in our dataset. The generalizability of our ensemble model to a broader array of IoT environments, each with its unique set of devices and traffic profiles, remains an area of consideration. Future research should aim to explore the model’s adaptability to various IoT ecosystems, encompassing a wider spectrum of devices, network architectures, and usage scenarios. Additionally, the discussion around device vulnerabilities warrants further elaboration. While our study briefly touches upon the notion of device vulnerabilities influencing the observed performance, a more in-depth analysis of specific vulnerabilities and their potential impact on the model’s effectiveness is imperative. Understanding the limitations imposed by device-specific vulnerabilities is essential for refining and tailoring our approach to address real-world security challenges more effectively.

Another aspect that merits attention is the scalability of our ensemble model. As IoT ecosystems continue to expand, accommodating an increasing number of devices, the scalability of intrusion detection systems becomes paramount. We acknowledge that the computational demands of our proposed approach may pose challenges in large-scale deployments. Future work should explore optimization strategies to enhance the scalability of the ensemble averaging DNN, ensuring its practical viability in extensive and dynamic IoT environments. Furthermore, the nature of our simulated environment, though valuable for controlled experimentation, introduces a degree of abstraction from real-world complexities. Factors such as dynamic network conditions, varying device behaviors over time, and the introduction of new IoT devices may present challenges not fully captured in our simulation. Future research could incorporate more dynamic and realistic settings to validate the robustness and adaptability of our model in real-world IoT scenarios. In conclusion, while our study contributes valuable insights into the effectiveness of ensemble averaging DNNs for botnet attack detection across heterogeneous IoT devices, we acknowledge these limitations and encourage ongoing research efforts to address them. A commitment to addressing these challenges will undoubtedly strengthen the reliability and practical utility of our proposed approach in the broader landscape of IoT security.

In future research, the exploration of the unique characteristics of individual IoT devices in more detail is needed. While our current study has highlighted differences in how traffic behaves among various devices, we haven’t thoroughly investigated the specific features that cause these distinctions. Moving forward, future research can delve deeper into understanding the particular attributes associated with each device. This deeper exploration could provide valuable insights into the distinct traits of individual devices, ultimately contributing to a more thorough understanding of the IoT landscape.

## Conclusion

This study aimed to devise an ensemble averaging DNN methodology for detecting botnet activities within a diverse IoT setting characterized by heterogeneous devices. The ensemble averaging DNN demonstrated a notable detection rate, showcasing a high level of accuracy compared to employing individual DNN models from each device to detect botnet attacks across the range of other devices. While the ensemble averaging DNN’s performance may fall short of that exhibited by individual models from each device when scrutinizing device-specific traffic, it shines when analyzing botnet attacks occurring across the spectrum of devices. This signifies that the proposed model possesses a comprehensive outlook, effectively analyzing and identifying botnet attacks within IoT environments featuring a variety of heterogeneous devices.

## Data Availability

The data that support the findings of this study are available to the public at the link: https://archive.ics.uci.edu/dataset/442/detection+of+IoT+botnet+attacks+n+baIoT.

## Code availability

The source code is available to the public at the link: https://github.com/aulwardana/ensemble-DNN-IoT.
